# Evaluation of perioperative prophylaxis with fosfomycin tromethamine in ureteroscopic stone removal: an investigator-driven prospective, multicenter, randomized, controlled study

**DOI:** 10.1007/s11255-017-1776-7

**Published:** 2017-12-30

**Authors:** Lu-Dong Qiao, Shan Chen, Yun-Hua Lin, Jian-Xing Li, Wei-Guo Hu, Jian-Ping Hou, Liang Cui

**Affiliations:** 1 Department of Urology, Beijing Tongren Hospital, Capital Medical University, No. 1 Dongjiaominxiang, Dongcheng District, 100730 Beijing, China; 2 Department of Urology, Beijing Anzhen Hospital, Beijing, China; 3 Department of Urology, Beijing Tsinghua Changgung Hospital, Beijing, China; 4 Department of Urology, Liangxiang Hospital of Capital Medical University, Yanjing Medical College, Beijing, China; 5 Department of Urology, Civil Aviation General Hospital, Beijing, China

**Keywords:** Antibiotic prophylaxis, Fosfomycin tromethamine, Ureteroscopy, Postoperative complications, Cost-effectiveness ratio

## Abstract

**Purpose:**

To compare efficacy, safety, and cost-effectiveness of fosfomycin tromethamine with other standard-of-care antibiotics in patients undergoing ureteroscopic lithotripsy.

**Methods:**

This study was a prospective, multicenter, randomized, controlled trial. Eligible patients scheduled for ureteroscopic lithotripsy were randomly assigned to receive either fosfomycin (fosfomycin group, *N* = 101 patients) or standard-of-care antibiotic therapy as prophylaxis (control group, *N* = 115 patients). The incidence of infectious complications and adverse events was analyzed between the two groups, as well as the cost–benefit analysis.

**Results:**

The incidence of infections following lithotripsy was 3.0% in the fosfomycin group and 6.1% in the control group (*p* > 0.05). Only asymptomatic bacteriuria was reported in fosfomycin group. In the control group was reported asymptomatic bacteriuria (3.5%), fever (0.9%), bacteremia (0.9%), and genitourinary infection (0.9%). The rate of adverse events was very low, with no adverse event reported in the fosfomycin group and only one in the control group (forearm phlebitis). The average cost per patient of antibiotic therapy with fosfomycin was 151.45 ± 8.62 yuan (22.7 ± 1.3 USD), significantly lower compared to the average cost per patient of antibiotics used in the control group 305.10 ± 245.95 yuan (45.7 ± 36.9 USD; *p* < 0.001).

**Conclusions:**

Two oral doses of 3 g fosfomycin tromethamine showed good efficacy and safety and low cost in perioperative prophylaxis of infections following ureteroscopic stone removal.

## Introduction

Due to crossover of antireflux barrier of the ureter, ureteroscopic procedures, including ureteroscopic stone removal, are associated with an increased risk of dissemination of pathogens from lower urinary tract to the upper tract and thus with an increased risk of post-procedure infections [1, 2]. Currently, there is consensus that patients undergoing ureteroscopic stone removal should receive antibiotic prophylaxis, which has been shown to reduce the incidence of post-procedure urinary tract infections [3–5]. While consensus exists on the need for antibiotic prophylaxis, guidelines on periprocedural prophylaxis in urological interventions recommend either fluoroquinolones or trimethoprim–sulfamethoxazole as first-line therapy [3] or to choose antibiotic regimen based on local epidemiology of drug resistance of potential pathogens [4] or based on the presence of other risk factors for infection and stone localization [6].

In China, the most frequently used antibiotics for the treatment of urinary tract infections (UTIs) and their prophylaxis post-endourologic procedures have been cephalosporins and fluoroquinolones [7]. The emergence of resistant strains, including strains producing extended-spectrum β-lactamase (ESBL), and safety issues related to fluoroquinolones has increased the interest in finding other antibiotics that can be used in UTI prophylaxis and therapy [3, 8]. Recently, fosfomycin has become an interesting alternative for treating UTIs due to the low prevalence of resistance of pathogens to this antibiotic [9].

Although the experience with fosfomycin for treatment of UTIs is growing in China, no data are yet available on its use for prophylaxis of infections post-ureteroscopic stone removal. In this context, we aimed to assess the efficacy and safety of fosfomycin tromethamine in the prophylaxis of infectious complications post semi-rigid ureteroscopic lithotripsy.

## Materials and methods

### Study design and participants

This was a prospective, multicenter, randomized, controlled trial performed between September 2015 and September 2016, in five hospitals in Beijing, China. Eligible patients were randomly assigned 1:1 to receive either fosfomycin tromethamine as preoperative prophylaxis (fosfomycin group) or other antibiotics according to local standard of practice (second-generation cephalosporin, fluoroquinolones, and other intravenous antibiotics; control group). The randomization was performed using a computer-generated list of random numbers.

All patients underwent transurethral retrograde semi-rigid ureteroscopic lithotripsy (holmium–YAG laser lithotripsy or pneumatic lithotripsy), and ureteral stent and catheter were routinely retained after operation. Four study visits were performed in all patients: baseline (Visit 1, before the ureteroscopic lithotripsy procedure), ureteroscopic lithotripsy procedure (Visit 2), 1–3 days following the procedure (Visit 3), and 1–4 weeks following the procedure (Visit 4). Patient data and characteristics, including medical history, biochemistry test results, and urine culture, were recorded at baseline. For the assessment of efficacy, urine test and urine culture were repeated at Visit 3 after catheter removal (1–3 day after the procedure) and at Visit 4 after double-J stent removal (1–4 week after ureteroscopic lithotripsy procedure), and body temperature was monitored. If body temperature was > 38 °C, blood culture was also performed. For the assessment of stone-free status (defined as no stone or stone fragments ≤ 4 mm), each patient underwent a kidney, ureter, and bladder X-ray investigation in the first day after ureteroscopic lithotripsy procedure and an urinary tract computer tomography scan before the double-J stent removal. Safety data, including assessment of kidney and liver function tests, were collected throughout the study.

Enrolled patients had uncomplicated ureteral stone, no or mild hydronephrosis, negative nitrites, and < 5 white blood cells/high power field at urine test. Ureteral stone was to be located either in the lower or middle ureter or in the upper ureter after shock wave lithotripsy (SWL) failure or patients had residual stones after SWL. Additionally, patients with radiolucent ureteral calculus were also an indication in our group. The main exclusion criteria were: kidney stone; moderate or severe hydronephrosis; positive urine culture [> 100,000 colony-forming units/ml (CFU/ml)]; allergy to the test drug; fever (≥ 38 °C); patients with preexisting ureteric stent or nephrostomy tube; use of immunosuppressants or medical history of immunodeficiency; liver disease with alanine transaminase and aspartate aminotransferase greater than twice the upper normal limit; renal dysfunction (creatinine level greater than 1.5 folds the upper normal limit); use of antibiotics within 72 h before enrollment; history of central nervous system or psychiatric disorders.

### Study treatment

Fosfomycin group received two oral doses of 3 g fosfomycin tromethamine powder administered 2–4 h before the ureteroscopic lithotripsy and in the morning following the procedure. Fosfomycin tromethamine was produced by Shanxi Qianyuan Pharmaceutical Co., Ltd (batch number 20150803) and was purchased locally by each participating institution. Control group received two doses of intravenous antibiotics, according to local standards—the first one administered 0.5 h before the procedure and the second one in the morning following the ureteroscopic lithotripsy.

### Primary and secondary endpoints

The primary endpoint was the incidence of infectious complications during the study, including asymptomatic bacteriuria (> 100,000 CFU/ml, no symptoms of infection), symptomatic UTIs (midstream voided urine culture > 10,000 CFU/ml with concomitant dysuria, urinary frequency, and urgency), fever (> 38 °C, excluding other infectious diseases), urosepsis, bacteremia, and genitourinary infection. Urosepsis was defined as the presence of at least 2 of the following: a temperature > 38 or < 36 °C, a pulse rate > 90 beats/min, a respiratory rate > 20 breaths/min or hypoventilation with a partial pressure of arterial carbon dioxide < 32 mmHg, and white blood cell count > 12,000 or < 4000 or ≥ 10% immature white blood cells [10]. Genitourinary infection in men was diagnosed if epididymitis and pain and swelling of the testicles were present. The secondary endpoints included safety and cost-effectiveness analyses.

### Statistical methods

Based on previous complication rates following endourological procedures reported in the literature [3–5], we assumed that in our sample complication rates will be of around 10%. Considering a non-inferiority margin of 0.05 (5%), a probability of type I error (α) of 0.025, and a confidence interval of 95%, we calculated that 229 patients should be enrolled. To account for an estimated dropout rate of 5%, we decided to enroll 240 patients.

The analysis of baseline data was to be performed in the full analysis set including all randomized patients, regardless of the number of antibiotic doses or study visits performed. Efficacy analysis was to be performed in per-protocol set (including those patients who completed the treatment originally allocated) and safety analysis in the safety analysis set (according to the treatment received and including those patients for which at least one safety assessment was performed).

Continuous data are shown as mean ± standard deviation and categorical variables as frequency. *t* test and Chi-square tests were used to compare data between study groups. Cost-effectiveness and sensitivity analyses were performed to assess the optimal balance between the cost and the effect of the study treatment. The cost-effectiveness ratio (*C*/*E*) represents the cost of per unit effect, with smaller values for lower costs. The sensitivity analysis was performed to evaluate the changes in the cost-effectiveness analysis when the cost varies. In this study, the cost was cut down by 10%. Data analysis was performed by using SPSS version 10.0 software. Statistical significance was defined as *p* < 0.05.

## Results

Two hundred and forty patients were enrolled and randomized in the two study groups (120 patients in each study group); of these 24 were excluded from the analysis due to noncompliance with main inclusion criteria. Thus, 216 patients fulfilling all inclusion criteria and without exclusion criteria (101 patients in fosfomycin group and 115 in control group) were included in the analysis presented here. All patients received treatments according to study groups they were randomized in and undergone all study procedures and visits. Therefore, all study populations were identical and included 216 patients.

Gender, age, frequency of mild preoperative hydronephrosis, stone size, and stone location were similar in the two study groups (Table 1). As per inclusion criteria (urine test and urine culture), none had urinary tract infection before ureteroscopic procedure. In the control group, the most frequently prescribed antibiotics were cephamycin (35.7% of the study group) and second-generation cephalosporins (31.7%). Other antibiotics prescribed were: penicillin (14.7%), fluoroquinolones (8.7%), and third-generation cephalosporin plus β-lactamase inhibitors (8.7%). As procedure for ureteral stone fragmentation, YAG laser lithotripsy was used in 52 (51.5%) patients in the fosfomycin group and 58 (50.4%) patients in the control group (*p* for the difference between groups > 0.05). Pneumatic lithotripsy was used in 49 (48.5%) patients in the fosfomycin group and 57 (49.6%) patients in the control group (*p* > 0.05). The length of the procedure and the stage I stone-free rates were also similar in the study groups: length of the procedure 35.4 ± 28.7 min versus 39.8 ± 27.8 min (*p* > 0.05); stage I stone-free rates 94% versus 92%, respectively (*p* > 0.05). There was no statistical difference between groups in the mean time of ureteral stent removal following the procedure: 15.5 ± 7.2 days in the fosfomycin group and 16.5 ± 6.8 days in the control group, *p* > 0.05.

**Table 1:** Baseline demographic and clinical characteristics

Characteristics	Fosfomycin group (*n* = 101)	Control group (*n* = 115)	*p* value
Age (years), mean ± SD	49.3 ± 15.2	47.7 ± 13.9	0.546
Male gender, *n* (%)	50 (49.5%)	65 (56.5%)	0.900
Stone location, *n* (%)			
Upper	26 (25.7%)	30 (26.1%)	0.421
Middle	46 (45.5%)	52 (45.2%)	
Lower	29 (28.7%)	33 (28.7%)	
Stone size, *n* (%)			
< 0.6 cm	12 (11.9%)	15 (13.0%)	0.959
0.6–0.8 cm	36 (35.6%)	42 (36.5%)	
0.8–1.2 cm	38 (37.6%)	40 (34.8%)	
> 1.2 cm	15 (14.9%)	18 (15.7%)	
Mild hydronephrosis, *n* (%)	65 (64.4%)	70 (60.9%)	0.990

*SD* standard deviation; *n* (%), number (percentage) of patients

The incidence of infections following lithotripsy in all patients was 4.6% (10/216), including asymptomatic bacteriuria 3.2% (7/216), two episodes of fever in one patient 0.5% (1/216), urosepsis 0.5% (1/216), and genitourinary infection 0.5% (1/216). Per study group, only asymptomatic bacteriuria was reported in fosfomycin group (3.0% of the patients). In the control group was reported asymptomatic bacteriuria (3.5%), fever (0.9%), bacteremia (0.9%), and genitourinary infection (0.9%). There was no statistically significant difference between study groups with regard to the post-procedure infections (*p* > 0.05; Table 2). The microorganisms identified in the urine culture of those with asymptomatic bacteriuria were *Escherichia coli* (1 patient in fosfomycin group and 2 patients in control group), *Enterococcus faecalis* and *pseudomonas aeruginosa* (1 patient in fosfomycin group), *Enterococcus faecalis* alone (1 patient in control group), *Lactobacillus gasseri* (1 patient in fosfomycin group), and *Staphylococcus haemolyticus* (1 patient in control group). In patients with other infectious complication, urine cultures were negative.

**Table 2:** Efficacy analysis—the frequency of post-surgery complications

	Overall complications, *n* (%)	Asymptomatic bacteriuria, *n* (%)	Symptomatic urinary tract infections, *n* (%)	Fever, *n* (%)	Urosepsis, *n* (%)	Bacteremia, *n* (%)	Genitourinary infection, *n* (%)
Fosfomycin group (*n* = 101)	3 (3.0%)	3 (3.0%)	0	0	0	0	0
Control group (*n* = 115)	7 (6.1%)	4 (3.5%)	0	1 (0.9%)	0	1 (0.9%)	1 (0.9%)
*p* value	> 0.05	> 0.05	–	> 0.05	–	> 0.05	> 0.05

*n* (%), number (percentage) of patients

One adverse drug reaction (forearm phlebitis) was reported in one patient from the control group who received cefmetazole sodium. No adverse drug reactions were reported in patients from fosfomycin group.

The average cost of antibiotic therapy in fosfomycin group was 151.5 ± 8.6 yuan (22.7 ± 1.3 USD; ranging between 135.6 and 160.0 yuan) and in control group was 305.1 ± 246.0 yuan (45.7 ± 36.9 USD; ranging between 36.8 and 859.1 yuan; *p* value for difference between groups < 0.001). These costs did not include the cost of infusion set and sodium chloride solution. The cost-effectiveness ratio was 1.6 for fosfomycin group and 3.3 for control group. The result of sensitivity analysis was consistent with the cost-effectiveness result, as shown in Table 3.

**Table 3:** Cost-effectiveness analysis

	Cost (yuan)	Effectiveness^a^ (%)	*C*/*E*	Δ*C*/Δ*E*
Cost-effectiveness				
Fosfomycin group	151.5	97.0	1.6	− 49.3
Control group	305.1	93.9	3.3	
Sensitivity^b^				
Fosfomycin group	136.3	97.0	1.4	− 44.3
Control group	274.6	93.9	2.9	

*C*/*E*, cost-effectiveness ratio; Δ*C*/Δ*E*, incremental cost-effectiveness ratio

^a^Effectiveness = 1—overall complication rate

^b^Sensitivity analysis = 10% off cost-effectiveness ratio

## Discussions

In this study, two oral doses of fosfomycin tromethamine administered 2–4 h before and post-ureteroscopic lithotripsy showed similar efficacy and safety with lower cost, compared with other antibiotics used as standard of care in the control group.

The rates of post-lithotripsy infections were low (3.0% in the fosfomycin group and 6.1% in the control group) consistent with previous studies on prophylaxis of UTIs following endourological procedures, in which were reported complication rates of 1.8–25% [1, 2]. Data on the use of fosfomycin for the prophylaxis of UTIs following endourological stone removal interventions are limited. We could identify only two uncontrolled studies—one enrolling 30 patients undergoing extracorporeal SWL and uretheroscopy and another one enrolling 60 patients (30 undergoing percutaneous nephrostomic lithotripsy) [11, 12]. These studies reported good efficacy, similar to the one we observed in our patients. In one of these studies was reported significant bacteriuria in 6% of the patients [11], while in the second one negative urine cultures were reported in 95% of the patients immediately after the procedure and in 85% 2–3 weeks after the procedure [12].

The rate of adverse events observed in our study groups was very low, with no adverse event reported in the fosfomycin group and only one in the control group. These results are consistent with the pooled safety analysis of 12 clinical trials in which one oral dose of 3 g of fosfomycin tromethamine had good tolerability, with adverse events, mainly gastrointestinal symptoms, reported in 6.1% of patients [13]. Similar low rates of adverse events of 5.1% were reported in an open-label clinical study in which the efficacy of three doses of fosfomycin tromethamine was studied in Chinese population [8].

We chose fosfomycin tromethamine as test intervention due to its bactericidal activity and broad antibacterial spectrum, with activity against both Gram-positive and Gram-negative pathogens [14–16]. It inhibits the biogenesis of bacterial cell wall by inactivating the phosphoenolpyruvate transferase, and thus it has a unique target and rarely interacts with other antibiotics [14]. Fosfomycin is also not affected by cross-resistance issues and has low toxicity and few adverse reactions [14]. Fosfomycin tromethamine has excellent pharmacological properties of fosfomycin and overcomes the shortcomings of fosfomycin calcium, such as the low bioavailability and absorption rate. Previous study showed that a single oral dose of 3 g fosfomycin tromethamine reaches a peak urine concentration within 4 h and a urine concentration > 128 mg/L is maintained for 24–48 h which is enough to suppress many kinds of uropathogens [14].

In our study, we found several antibiotics used in the perioperative prophylaxis in the control group. Cephamycins were ranked first, and second-generation cephalosporins and penicillins antimicrobial second. Additionally, fluoroquinolones were chosen by urological surgeons in 8.7% of the patients in the control group. Although all antibiotics used in this study were effective, previous studies performed in China showed that antibiotic resistance of *E. coli* clinical isolates to fluoroquinolones and cephalosporins is on the rise [7, 17–19], suggesting limited efficacy of these. Both cephalosporins and fluoroquinolones remain the most commonly used antibiotics in China, due to their inclusion in the current guidelines and due to consensus that some antibiotics can be used for prophylaxis despite increasing resistance rates to preserve the sensitivity to other antibiotics used in the therapy. Meanwhile, no increases in *E. coli* strains resistance to fosfomycin have been reported [17, 20]. In China, fosfomycin tromethamine has been shown to have good antibacterial activity against both ESBL producing and ESBL-negative *E. coli*, with resistance rates of 4.3 and 0%, and bacterial sensitivity rates of 87.0 and 95.1%, respectively [17].

The cost analysis showed that the average cost per patient of antibiotic therapy with fosfomycin was 22.7 USD, significantly lower compared to the average cost per patient of other antibiotic drugs used (45.7 USD). Additionally, the cost-effectiveness ratio was significantly lower for fosfomycin group (1.6) than for control group (3.3).

A limitation of our study was the heterogenic group of antibiotics used as control. As currently there is no consensus on which antibiotic should be used in ureteroscopic procedures as prophylaxis, we also used this study to evaluate the current state of antibiotics used in the urological infections in China.

In conclusion, this is the first multicenter study to evaluate the perioperative prophylaxis with fosfomycin tromethamine in ureteroscopic stone removal by comparison with standard-of-care intravenous antibiotics in China, suggesting this regimen is safe and has good efficacy. Fosfomycin’s low local cost as compared to other antibiotics combined with increasing local resistance to other antibiotics makes fosfomycin a highly attractive and cost-effective option for the prophylaxis of infections following endourological procedures. Fosfomycin may represent a valid alternative to cephalosporin and fluoroquinolones for prophylactic purposes and thus may help preventing further increase in the resistance to these antibiotics. We believe that our study can be informative for physicians in their decision-making in the perioperative prophylaxis in ureteroscopic stone removal.
